# Short-term clinical outcomes of robotic-assisted total knee arthroplasty at 12-month follow-up: a prospective, multicenter, concomitant comparison to conventional total knee arthroplasty

**DOI:** 10.1007/s00402-026-06338-9

**Published:** 2026-05-06

**Authors:** Ulrich Nöth, Gurion Rivkin, Patrizio Caldora, Karl-Dieter Heller, Emmanuel Thienpont, Didier Hannouche, Itay Perets, Jason Cholewa

**Affiliations:** 1 Evangelisches Waldkrankenhaus Spandau, Berlin, Germany; 2 Hadassah Medical Center, Jerusalem, Israel; 3 San Giuseppe Hospital-Korian, Arezzo, Italy; 4 Herzogin Elisabeth Hospital, Braunschweig, Germany; 5 Cliniques Universitaires Saint-Luc, Brussels, Belgium; 6 ZAS-Cadix, Antwerpen, Belgium; 7 University Hospital of Geneva, Geneva, Switzerland; 8 Zimmer Biomet (United States), Warsaw, USA

**Keywords:** Arthroplasty, Replacement, Knee, Robotics, Treatment outcome, Prospective study, Satisfaction, Function

## Abstract

**Introduction:**

There is limited data available on short-term outcomes on a cut-block positioning robotic system. The purpose of this study was to compare 12-month clinical outcomes between robotic-assisted (raTKA) and conventional total knee arthroplasty (cTKA) with multiple outcomes and surgical centers.

**Methods:**

This was a non-randomized controlled trial of patients who received either raTKA (*n* = 120) or cTKA (*n* = 101) at 6 different surgical centers. Variables of interest included occurrence of soft tissue release, complications and revisions at minimum one-year follow-up. Satisfaction, pain (numeric rating scale [NRS]), 5-dimensional European Quality of Life (EQ-5D-5 L) questionnaire (index and visual analog scale [VAS]), Oxford Knee Score (OKS), and the Forgotten Joint Score (FJS-12) were collected pre-operatively, and at six weeks, three months, and 12 months post-operative.

**Results:**

There were significantly less soft tissue releases with raTKA (28/120, 23.3%) vs. cTKA (51/99, 51.5%), *p* < 0.0001). There were significantly fewer cases of medial/lateral instability in the raTKA group at six-weeks (*p* = 0.038) and three-months (*p* = 0.007) post-operative. At one-year follow-up, 96.3 and 92.5% of raTKA and cTKA patients were satisfied with the overall results of their surgery, respectively. Significantly more raTKA patients were very satisfied (32.1% vs. 14.6%) with their ability to do home/yard work at six weeks (*p* = 0.018). Significantly (*p* = 0.042) less raTKA patients were dissatisfied (5.1% vs. 12.9%) with their ability to perform recreation at one-year post-operative. The EQ-5D-5 L increased significantly (*p* = 0.042) more in the raTKA group at one-year post-operative (0.529 ± 0.335 vs. 0.417 ± 0.323), but did not exceed the minimal clinical important difference.

**Conclusion:**

raTKA was associated with fewer soft tissue release procedures and medial/lateral instability with greater satisfaction in performing home/yard work at six-weeks post-operative. raTKA was equivalent to cTKA for overall satisfaction, quality of life, and knee-specific patient reported outcome measures in the early post-operative period.

**Level of evidence:**

II.

## Introduction

Satisfaction is a multifactorial patient reported outcome measure (PROM) and influenced by non-modifiable factors, such as age and sex [1]. However, intra-operative modifiable factors related to component alignment, gap balance, soft tissue releases, and iatrogenic damage have also been reported to be related to patient satisfaction and function [2–7].

Robotic-assisted total knee arthroplasty (raTKA) technologies were introduced to provide surgeons with greater control and accuracy over bone preparation and alignment compared with conventional jig-based manual instrumentation (cTKA). A growing body of literature now provides evidence that raTKA is superior to cTKA for resection accuracy, implant positioning, limb alignment, gap balance, and periarticular tissue damage [8–18]. raTKA also appears a suitable technology to address difficult knees, including fixed flexion deformity [19], extra-articular deformity [20], and severe varus and valgus deformities [21]. Additionally, the ability to evaluate gap balance and laxity through the arc of range of motion intra-operatively prior to bony resections with raTKA may lessen the need for soft tissue releases. Collateral ligament releases have been estimated to be required in 50–86% of cTKA cases [22, 23], while recent studies report significantly lower rates of release at 13–43% of cases [22, 24] in raTKA. Though differences in knee alignment philosophy will influence the need for soft tissue releases [25], there is evidence to suggest a negative association between soft tissue release and post-operative function through two-years follow-up [22].

Greater accuracy and less soft tissue damage is hypothesized to lead to superior clinical outcomes with raTKA. However, the state of the literature related to clinical outcomes are presently ambiguous, with some [8, 26] but not all meta-analyses reporting superior PROMs with raTKA [27, 28]. These discrepancies in meta-analyses may be due to differences in the PROMs collected between studies (i.e.: Western Ontario and McMaster Universities Arthritis Index (WOMAC) vs. Knee Society Score (KSS) and the follow-up periods reported, thus limiting statistical power. Additionally, there is still a need to evaluate multicenter outcome data with larger sample sizes. Therefore, the purpose of this study was to compare early pre-operative clinical outcomes between raTKA and cTKA with multiple PROMs and surgical centers.

## Methods

**Fig. 1:**
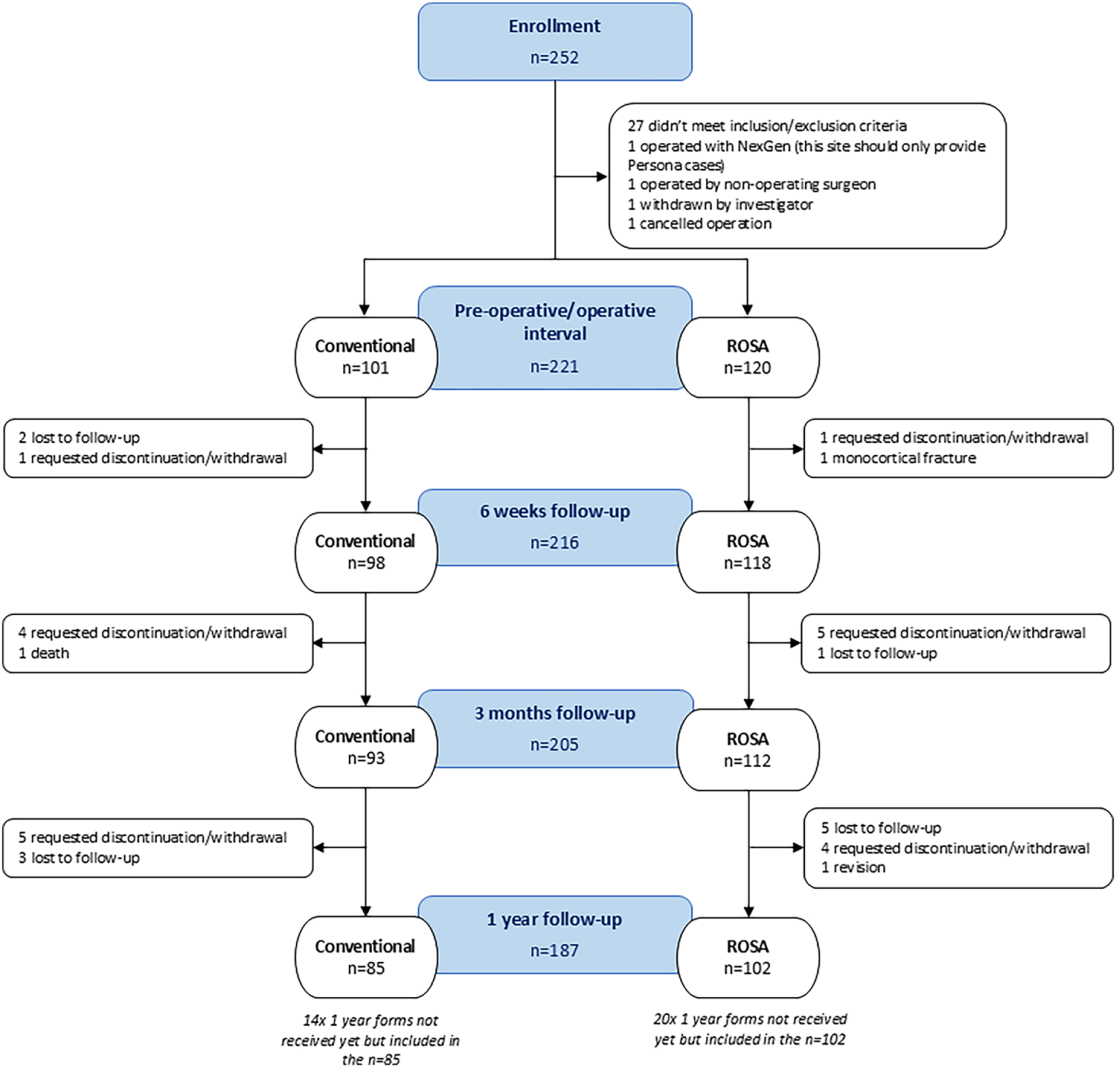
CONSORT flow diagram

This was a prospective, multi-center, cohort study of patients who received primary TKA between December 2020 and October 2022 by six surgeons each at a different surgical center in Europe (clinicaltrials.gov: NCT04338893). To avoid potential selection bias, participation was offered consecutively to eligible patients, and surgeons performed 5 raTKAs followed by 5 cTKAs using competitive enrollment until the maximum study enrollment of 240 patients was reached. Patients who qualified for a TKA were eligible for participation. Patients were excluded from participation if they were currently participating in any other surgical intervention or pain management studies, underwent contralateral partial or total knee arthroplasty within the last 18 months, had pathologies that could influence bone metabolism, had hip pathologies with significant bone loss or that severely limited range of motion, or had previously received a partial or total knee arthroplasty for the ipsilateral knee. Ethics approval for all surgical centers was obtained and all participants provided written informed consent prior to engaging in any study related procedures. A flow diagram of patients is presented in Fig. 1.

Patients in the raTKA group (ROSA^®^ Knee System, Zimmer Biomet, Montreal, Quebec, Canada) and cTKA group received either the Persona^®^ Knee System (Zimmer Biomet, Warsaw, IN, USA) or Vanguard^®^ Knee System (Zimmer Biomet, Warsaw, IN, USA) implants. Standard surgical procedure was followed under general or spinal anesthesia according to the surgeon’s preference. Table 1 describes the alignment philosophy of each surgeon for raTKA and cTKA. Surgical data collected included pre-operative diagnosis, surgical approach, intra-operative complications, additional procedures, number of trays, surgical time, and the polyethylene bearing used.

**Table 1:** Surgeon TKA alignment philosophies

Surgeon 1	raTKA	mTKA
	Functional alignment	Mechanical alignment
Surgeon 2	Functional alignment	N/A
Surgeon 3	Functional alignment	Restricted kinematic alignment
Surgeon 4	Adjusted mechanical alignment	Adjusted mechanical alignment
Surgeon 5	Reverse kinematic alignment	Mechanical alignment
Surgeon 6	Mechanical alignment	Mechanical alignment

The outcomes collected in this study included flexion range of motion (ROM), a numeric rating scale (NRS) for pain [29], the 5-dimensional European Quality of Life (EQ-5D-5 L) questionnaire (index and visual analog scale [VAS]), the Oxford Knee Score (OKS), and the Forgotten Joint Score (FJS-12). The minimal clinical important difference (MCID) has been reported as 2.0, 0.26, 5.0, and 16.6 for the NRS pain [30, 31], EQ-5D-5 L [32], OKS [33], and FJS [34], respectively. Objective knee indicators from the 2011 Knee Society Score, including medial/lateral instability (none, little or < 5 mm, moderate or 5 mm, severe or > 5 mm) and anterior/posterior instability (none, moderate < 5 mm, severe > 5 mm) [35] were collected. All follow-up measures were assessed pre-operatively and at six weeks, three- and twelve-months post-operative.

A four item (How satisfied are you with the results of your surgery? How satisfied are you with the results of your surgery for improving pain? How satisfied are you with the results of your surgery for improving your ability to do home or yard work? How satisfied are you with the results of your surgery for improving your ability to do recreational activities?), 4-point (Very Satisfied, Somewhat Satisfied, Somewhat Dissatisfied, Very Dissatisfied), Likert scale for satisfaction was administered at six weeks, three- and twelve-months post-operative. We also reviewed any adverse events associated with the surgical limb through one-year post-operative.

### Statistical analysis

All data is presented as means ± standard deviations, unless otherwise noted. Categorical data was analyzed with Fisher’s exact test or Pearson’s Chi Square test where appropriate. Continuous data was analyzed with independent samples *t*-tests without adjustment for multiplicity. Statistical significance was set to an α of *p* < 0.05 a priori, and all analyses were performed with SAS v9.4 (SAS Institute, Inc. Cary, NC, USA).

## Results

There were no significant (*p* > 0.05) differences between groups in patient demographics, pre-operative diagnoses, type of anesthesia, surgical approach, or type of component used (Table 2). Surgeons contributed approximately 3 fewer raTKA procedures (16.9 ± 9.6) than cTKA (20 ± 8.7) procedures to the study cohort.

**Table 2:** Pre-operative patient characteristics

	cTKA (*n* = 101)	raTKA (*n* = 120)
Height (cm: mean ± SD)	167.1 *±* 9.2	167.1 *±* 9.1
Weight (kg: mean ± SD)	82.9 *±* 15.0	82.8 *±* 14.3
Body Mass Index (m/kg^2^: mean ± SD)	29.7 *±* 5.1	29.7 *±* 4.8
Age (mean ± SD)	70.4 *±* 8.7	70.6 *±* 7.9
Sex (n female, %)	66/101, 65.3%	75/120, 62.5%
Osteoarthritis (n, %)	94/101, 93.1%	114/120, 95.0%
Surgical approach (n, %)		
Medial parapatellar	82/99, 81.2%	99/120, 82.5%
Midvastus	3/99, 2.8%	N/A
Subvastus	11/99, 10.9%	20/120, 16.7%
Lateral parapatellar	1/99 1.0%	1/120, 0.8%
Lateral	2/99, 2.0%	N/A
Missing	2/99, 2.0%	
Anesthesia (n, %)		
General	50/101, 49.5%	63/120, 52.5%
Spinal	42/101, 41.6%	46/120, 38.3%
Multimodal	8/101, 7.9%	11/120, 9.2%
Peripheral Nerve Block	1/101, 1.0%	N/A
Component^*^ (n, %)		
Persona CR cemented	13/101, 12.9%	13/120, 10.8%
Persona CR cementless	1/101, 1.0%	N/A
Persona PS cemented	54/101, 53.4%	73/120, 60.8%
Persona PS cementless	3/101, 3.0%	6/120, 5.0%
Vanguard CR cemented	21/101, 20.8%	19/120, 15.8%
Vanguard PS cemented	9/101, 8.9%	8/120, 6.7%
Patellar resurfacing (n, %)	27/101, 26.7%	31/120, 25.8%

^*^CR Cruciate retaining, *PS* Posterior stabilized

There were significantly (*p* < 0.0001) more soft tissue releases performed in cTKA (51 cases, 51.5%) compared to raTKA (28 cases, 23.3%) (Table 3). The odds of requiring a soft tissue release for cTKA vs. raTKA was 3.49 (95% CI: 1.97, 6.29).

**Table 3:** Occurrence of soft tissue releases performed^a^

Release	cTKA (*n*, %)	rTKA (*n*, %)	*p* value (OR: 95% CI)
None	48/99, 48.5%	28/120, 76.7%	< 0.0001 (0.32: 0.18, 0.58)
Medial	29/99, 29.3%	15/120, 12.5%	0.0023 (0.34: 0.17, 0.69)
Lateral retinacular	17/99, 17.2%	9/120, 7.5%	0.0345 (0.39: 0.17, 0.92)
Posterior capsule	9/99, 9.1%	8/120, 6.7%	0.4543 (0.71: 0.26, 1.93)
Illiotibial band	11/99, 11.1%	5/120, 4.2%	0.0583 (0.35: 0.12, 1.04)
Posterior lateral	6/99, 6.1%	4/120, 3.3%	0.3533 (0.53: 0.15, 1.95)
Superficial medial collateral ligament	5/99, 5.1%	2/120, 1.7%	0.248 (0.32: 0.06, 1.68)
Popliteus tendon	1/99, 1%	2/120, 1.7%	0.9999 (1.66: 0.15, 18.60)
Lateral collateral ligament	0/99, 0%	1/120, 0.8%	0.9999 (2.50: 0.11, 62.00)

^a^Fishers exact test

There were no significant (*p* > 0.05) differences between groups for overall satisfaction with the results of the surgery nor satisfaction with the results of the surgery for improving pain at six weeks, three months, or 12 months post-operative (Table 2). Significantly more raTKA patients were very satisfied with their ability to do home/yard work at six weeks (*p* = 0.018). There were also significant (*p* = 0.042) differences found for satisfaction with the ability to perform recreational activities at twelve-months, where 7% more cTKA patients were very satisfied, while 15% more raTKA patients were somewhat satisfied and 7% less raTKA patients were somewhat dissatisfied (Table 4).

**Table 4:** Patient satisfaction scores^a^

Time	Outcome	cTKA (*n*, %)	raTKA (*n*,%)	*P* value
Item 1	Satisfied with the results of your surgery			
6 Weeks	Very Satisfied	49/89, 55.1%	54/106, 50.9%	0.6261
	Somewhat Satisfied	29/89, 32.6%	42/106, 39.6%	
	Somewhat Dissatisfied	10/89, 11.2%	8/106, 7.5%	
	Very Dissatisfied	1/89, 1.1%	2/106, 1.9%	
3 Months	Very Satisfied	48/88, 54.5%	62/104, 59.6%	0.5927
	Somewhat Satisfied	33/88, 37.5%	35/104, 33.7%	
	Somewhat Dissatisfied	4/88, 4.5%	6/104, 5.8%	
	Very Dissatisfied	3/88, 3.4%	1/104, 1.0%	
12 Months	Very Satisfied	42/70, 60.0%	48/81, 59.3%	0.7311
	Somewhat Satisfied	23/70, 32.9%	30/81, 37.0%	
	Somewhat Dissatisfied	4/70, 5.7%	3/81, 3.7%	
	Very Dissatisfied	1/70, 1.4%	0/81, 0.0%	
Item 2	Satisfied with the results of your surgery for improving pain			
6 Weeks	Very Satisfied	31/89, 34.8%	41/106, 38.7%	0.2736
	Somewhat Satisfied	43/89, 48.3%	51/106, 48.1%	
	Somewhat Dissatisfied	15/89, 16.9%	11/106, 10.4%	
	Very Dissatisfied	0/89, 0.0%	3/106, 2.8%	
3 Months	Very Satisfied	49/88, 55.7%	63/103, 61.2%	0.2106
	Somewhat Satisfied	31/88, 35.2%	31/103, 30.1%	
	Somewhat Dissatisfied	5/88, 5.7%	9/103, 8.7%	
	Very Dissatisfied	3/88, 3.4%	0/103, 0.0%	
12 Months	Very Satisfied	50/70, 71.4%	49/81, 60.5%	0.1501
	Somewhat Satisfied	13/70, 18.6%	27/81, 33.3%	
	Somewhat Dissatisfied	4/70, 5.7%	4/81, 4.9%	
	Very Dissatisfied	3/70, 4.3%	1/81, 1.2%	
Item 3	Satisfied with the results of your surgery for improving the ability to do home/yard work			
6 Weeks	Very Satisfied	13/89, 14.6%	34/106, 32.1%	0.0178
	Somewhat Satisfied	60/89, 67.4%	57/106, 53.8%	
	Somewhat Dissatisfied	15/89, 16.9%	12/106, 11.3%	
	Very Dissatisfied	1/89, 1.1%	3/106, 2.8%	
3 Months	Very Satisfied	33/88, 37.5%	56/104, 53.8%	0.0996
	Somewhat Satisfied	42/88, 47.7%	40/104, 38.5%	
	Somewhat Dissatisfied	8/88, 9.1%	6/104, 5.8%	
	Very Dissatisfied	5/88, 5.7%	2/104, 1.9%	
12 Months	Very Satisfied	35/70, 50.0%	45/80, 56.3%	0.2685
	Somewhat Satisfied	26/70, 37.1%	30/80, 37.5%	
	Somewhat Dissatisfied	9/70, 12.9%	4/80, 5.0%	
	Very Dissatisfied	0/70, 0.0%	1/80, 1.3%	
Item 4	Satisfied with the results of your surgery for improving the ability to do recreational activities			
6 Weeks	Very Satisfied	17/89, 19.1%	34/106, 32.1%	0.2096
	Somewhat Satisfied	52/89, 58.4%	54/106, 50.9%	
	Somewhat Dissatisfied	16/89, 18.0%	14/106, 13.2%	
	Very Dissatisfied	4/89, 4.5%	4/106, 3.8%	
3 Months	Very Satisfied	37/88, 42.0%	55/104, 52.9%	0.3226
	Somewhat Satisfied	36/88, 40.9%	37/104, 35.6%	
	Somewhat Dissatisfied	10/88, 11.4%	10/104, 9.6%	
	Very Dissatisfied	5/88, 5.7%	2/104, 1.9%	
12 Months	Very Satisfied	43/70, 61.4%	43/79, 54.4%	0.0416
	Somewhat Satisfied	18/70, 25.7%	32/79, 40.5%	
	Somewhat Dissatisfied	9/70, 12.9%	3/79, 3.8%	
	Very Dissatisfied	0/70, 0.0%	1/79, 1.3%	

^a^Fishers exact test

**Fig. 2:**
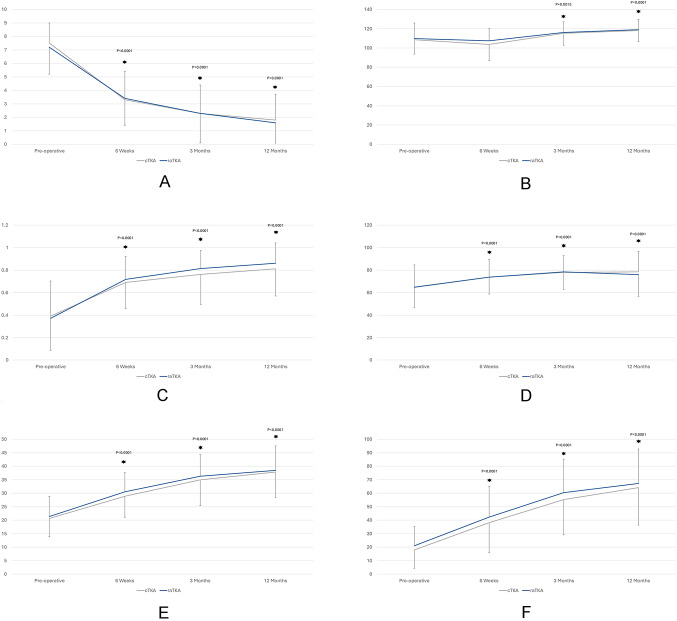
Outcome measures for NRS Pain Score (**A**), Flexion Range of Motion (**B**), EuroQol 5-Dimension-5-Level (**C**), EuroQol Visual Analogue Scale (**D**), Oxford Knee Score (**E**), and Forgotten Joint Score (**F**).*significantly different from pre-operative

Both groups significantly improved from pre-operative to three- and twelve-months post-operative in NRS pain, ROM, EQ-5D-5 L, EQ VAS, OKS, and FJS without significant (*p* > 0.05) differences between groups (Fig. 2A-F). When comparing the change from baseline, there were significant (*p* = 0.042) differences in EQ-5D-5 L favoring raTKA at twelve-months post-operative (Table 5). There were no differences between groups in the frequency of patients achieving an MCID for NRS pain, EQ-5D-5 L, OKS, or FJS (Table 6).

**Table 5:** Difference from pre-operative values for outcome measures

Time	Cohort (*n*)	Change (mean ± sd (95% C.I.))	*P* Value
NRS pain score			
6 Week	cTKA (89)	−4.1 ± 2.3 (−4.6, −3.7)	0.4696
	raTKA (106)	−3.9 ± 2.3 (−4.4, −3.5)	
3 Month	cTKA (88)	−5.1 ± 2.3 (−5.6, −4.6)	0.8421
	raTKA (104)	−5.0 ± 3.0 (−5.6, −4.5)	
1 Year	cTKA (70)	−5.9 ± 2.1 (−6.4, −5.4)	0.8379
	raTKA (81)	−5.9 ± 2.7 (−6.5, −5.3)	
Flexion range of motion			
6 Week	cTKA (89)	−5.0 ± 20.0 (−9.2, −0.8)	0.4457
	raTKA (106)	−2.8 ± 20.3 (−6.7, 1.1)	
3 Month	cTKA (85)	5.9 ± 16.5 (2.3, 9.4)	0.803
	raTKA (104)	5.2 ± 17.8 (1.8. 8.7)	
1 Year	cTKA (66)	9.1 ± 16.9 (4.9, 13.3)	0.6825
	raTKA (76)	10.3 ± 19.1 (6.0, 14.7)	
EQ-5D-5 L			
6 Week	cTKA (89)	0.278 ± 0.298 (0.214, 0.343)	0.0982
	raTKA (106)	0.355 ± 0.329 (0.291, 0.419)	
3 Month	cTKA (88)	0.351 ± 0.340 (0.277, 0.425)	0.0628
	raTKA (104)	0.447 ± 0.353 (0.378, 0.517)	
1 Year	cTKA (70)	0.417 ± 0.323 (0.337, 0.496)	0.0417
	raTKA (81)	0.529 ± 0.335 (0.455, 0.604)	
EQ VAS health state			
6 Week	cTKA (89)	9.0 ± 22.0 (4.3, 13.8)	0.9107
	raTKA (106)	9.4 ± 19.1 (5.7, 13.1)	
3 Month	cTKA (88)	12.4 ± 18.2 (8.5, 15.4)	0.6338
	raTKA (104)	13.8 ± 19.4 (9.9, 17.6)	
1 Year	cTKA (70)	14.3 ± 23.2 (8.6, 20.0)	0.4807
	raTKA (80)	11.6 ± 21.9 (6.7, 16.5)	
Oxford knee score			
6 Week	cTKA (89)	8.4 ± 8.8 (6.5, 10.3)	0.543
	raTKA (106)	9.2 ± 9.1 (7.4, 11.0)	
3 Month	cTKA (88)	14.1 ± 10.6 (11.8, 16.4)	0.6288
	raTKA (104)	14.9 ± 10.0 (12.9, 16.8)	
1 Year	cTKA (70)	17.2 ± 10.2 (14.7, 19.7)	0.7627
	raTKA (80)	16.7 ± 10.1 (14.4, 18.9)	
Forgotten joint score			
6 Week	cTKA (89)	19.1 ± 23.4 (14.0, 21.1)	0.5693
	raTKA (106)	21.0 ± 23.8 (16.4, 25.6)	
3 Month	cTKA (88)	37.3 ± 28.0 (31.3, 43.4)	0.5972
	raTKA (103)	39.4 ± 26.0 (34.3, 44.6)	
1 Year	cTKA (69)	47.2 ± 27.9 (40.3, 54.1)	0.9839
	raTKA (80)	47.1 ± 26.5 (41.2, 53.1)	

**Table 6:** Percentage of patients achieving an MCID

Time	cTKA	raTKA	*P* Value
NRS pain score improvement from pre-operative MCID = 2			
6 Week (n,%)	82, 92.1%	90, 84.9%	0.1802
3 Month (n,%)	82, 93.2%	91, 87.5%	0.2298
1 Year (n,%)	70, 100%	77, 95.1%	0.124
EQ-5D-5 L score improvement from pre-op MCID = 0.26			
6 Week (n,%)	44, 52.4%	57, 54.3%	0.8835
3 Month (n,%)	56, 66.7%	68, 66.7%	1.000
1 Year (n,%)	48, 72.7%	64, 80.0%	0.33
Oxford knee score improvement from pre-op MCID = 5			
6 Week (n,%)	55, 65.5%	70, 67.3%	0.8767
3 Month (n,%)	68, 81.0%	84, 82.4%	0.8502
1 Year (n,%)	56, 84.9%	68, 86.1%	0.999
Forgotten joint score improvement from pre-op MCID = 16.6			
6 Week (n,%)	47, 56.0%	57, 54.3%	0.8833
3 Month (n,%)	64, 76.2%	81, 80.2%	0.5913
1 Year (n,%)	54, 83.1%	69, 87.3%	0.4871

There were significant (*p* = 0.0406) differences in pre-operative anterior/posterior instability, with approximately 10% more cases of moderate instability in the raTKA cohort and 6% more cases of severe instability in the cTKA cohort, but no differences between cohorts at any post-operative assessment (Table 7). There were significant differences between cohorts at six-weeks (*p* = 0.038) and three-months (*p* = 0.007) post-operative, with approximately 10% and 16% more cTKA patients presenting with medial/lateral instability at the respective assessment times (Table 7).

**Table 7:** Objective knee assessment rates of instability^1^

Time	Instability	cTKA	raTKA	p value
Anterior/posterior instability				
Preop (% (n)	Moderate	15.80%	25.00%	0.0406
		(16/101)	(30/120)	
	None	75.20%	72.50%	
		(76/101)	(87/120)	
	Severe	8.90%	2.50%	
		(9/101)	(3/120)	
6 Week (% (n)	Moderate	11.20%	10.40%	0.7251
		(10/89)	(11/106)	
	None	87.60%	89.60%	
		(78/89)	(95/106)	
	Severe	1.10%	0.00%	
		(1/89)	(0/106)	
3 Month (% (n)	Moderate	15.30%	12.50%	0.2919
		(13/85)	(13/104)	
	None	82.40%	87.50%	
		(70/85)	(91/104)	
	Severe	2.40%	0.00%	
		(2/85)	(0/104)	
1 Year (% (n)	Moderate	13.60%	10.50%	0.637
		(9/66)	(8/76)	
	None	83.30%	88.20%	
		(55/66)	(67/76)	
	Severe	3.00%	1.30%	
		(2/66)	(1/76)	
Medial/lateral instability				
Preop (% (n)	Little	18.80%	26.70%	0.1972
		(19/101)	(32/120)	
	Moderate	17.80%	9.20%	
		(18/101)	(11/120)	
	None	58.40%	60.00%	
		(59/101)	(72/120)	
	Severe	5.00%	4.20%	
		(5/101)	(5/120)	
6 Week (% (n)	Little	14.60%	9.40%	0.0384
		(13/89)	(10/106)	
	Moderate	4.50%	0.00%	
		(4/89)	(0/106)	
	None	80.90%	90.60%	
		(72/89)	(96/106)	
3 Month (% (n)	Little	22.40%	8.70%	0.0073
		(19/85)	(9/104)	
	Moderate	3.50%	1.00%	
		(3/85)	(1/104)	
	None	74.10%	90.40%	
		(63/85)	(94/104)	
1 Year (% (n)	Little	18.20%	10.50%	0.1722
		(12/66)	(8/76)	
	Moderate	4.50%	1.30%	
		(3/66)	(1/76)	
	None	75.80%	88.20%	
		(50/66)	(67/76)	
	Severe	1.50%	0.00%	
		(1/66)	(0/76)	

^a^Fishers exact test

There were no significant (*p* = 0.720, OR: 0.87, 95% CI: 0.43, 1.76) differences in the occurrence of adverse events between raTKA (*n* = 19/120, 15.8%) and cTKA (*n* = 18/101, 17.8%) (Table 8). There was one case of periprosthetic joint infection at four months post-operative in the raTKA cohort that led to device removal. There were three adverse events in the raTKA group directly related to the robotic device: the first was a tibial hematoma at the pin site, the second was a malfunction leading to a tibial resection of 6 mm too deep, and one case of femoral periprosthetic joint fracture in the raTKA group near Fiche’s hole that resulted in study withdrawal at seven weeks post-operative. There were no cases of pin site fractures or aseptic loosening.

**Table 8:** Procedure and device related adverse events

Adverse event	raTKA (*n*)	cTKA (*n*)	*p* value (OR: 95% CI)
Patella mal-tracking	2	3	0.5424 (0.54: 0.09, 3.31)
Restricted mobility	7^a^	7^a^	0.7099 (0.81: 0.28, 2.41)
Persistent Effusion	3	3	0.8112 (0.82: 0.17, 4.16)
Vastus lateralis rupture	0	1	0.9999 (0.27: 0.01, 6.76)
Hematoma	1	0	0.9999 (2.45: 0.10, 62.00)
Periprosthetic joint fracture	1	0	0.9999 (2.45: 0.10, 62.00)
Retained cement	1	0	0.9999 (2.45: 0.10, 62.00)
Persistent pain	0	1	0.9999 (0.27: 0.01, 6.76)
Wound dissonance	1	2	0.5919 (0.41: 0.04, 4.57)
Deep vein thrombosis	1	1	0.8913 (0.82: 0.05, 13.34)
Robotic malfunction	1	0	0.9999 (2.45: 0.10, 62.00)
Periprosthetic joint infection	1	0	0.9999 (2.45: 0.10, 62.00)

^a^One case requiring manipulation under anesthesia

There were significant (*p* < 0.001) differences between groups for number of trays (cTKA: 6.4 *±* 1.7; raTKA: 6.9 *±* 1.5), time from incision cut to close (cTKA: 77.5 *±* 20.3; raTKA 91.5 *±* 21.1 min), and time under anesthesia (cTKA: 122.5 *±* 28.4; 142.0 *±* 27.1 min).

## Discussion

The primary findings of this study were that both cTKA and raTKA improved patient reported outcomes, however, more patients in the raTKA cohort were very satisfied with their ability to perform home and yard work (activities of daily living: ADLs) at six-weeks and reported greater improvements in quality of life at one-year post-operative. At one-year follow-up, approximately 93% of cTKA and 96% of raTKA patients were either somewhat or very satisfied with the results of their surgery. These findings are consistent with a recent paper that reported 95% of raTKA patients were happy they had surgery at a minimum one year follow-up [36].

Our findings related to higher satisfaction with the ability to perform ADLs suggest an earlier return to light activity with raTKA. Smith et al. [37] used a similar Likert-scale survey to assess satisfaction with the ability to perform ADLs and recreation and reported no difference between raTKA and cTKA at six weeks or one-year post-operative. Methodological differences may explain this discrepancy between studies, as Smith et al. separated the responses into either dissatisfied, neutral, or satisfied, and did not stratify between very satisfied or somewhat satisfied as we did. On the other hand, the insignificant findings related to the ability to perform recreational activities at six-week and six-month follow-up are likely due to limitations on, and the inability to perform, physical activity in the early post-operative period.

There were fewer intra-operative adjustments to release soft tissues in the raTKA cohort. The differences in releases between groups may be directly attributable to the robotic intervention. The robotic knee system provides real-time intra-operative planning of gap balance prior to any cuts as well as the assessment of flexion and extension gaps prior to final implantation, thereby allowing surgeons with a non-mechanical alignment philosophy to rely on boney resections and minimizing the need for soft tissue release to correct imbalances. Previous studies on non-mechanical alignment have reported robotic-assistance that increases tibial varus beyond 3° is not associated with greater risk of revision, complications or poorer functional outcomes [38], and balancing pre-operative varus deformity with slight varus alignment in flexion was associated with improved functional outcomes [39]. Given the inverse relationship between soft tissue release, recovery rates, and patient satisfaction [5, 22], this finding, in addition to greater medial/lateral stability at six-weeks and three-months post-operative, may have contributed to the greater magnitude of satisfaction with the ability to perform ADLs in the raTKA cohort. Similar to the findings in the present study, Clapp et al. [40] reported a lower overall incidence in soft tissue release procedures with raTKA (29.9%) compared to computer navigated (74.4%) or cTKA (47.9%). The authors also reported a greater reduction in pain and greater improvement in KOOS JR at six-weeks post-operative in the raTKA cohort compared to the computer navigated cohort.

Improvements in pain, OKS, KSS, FJS, and EQ-5D-5 L were observed overtime, with mean change scores first exceeding their respective MCIDs at three-months post-operative. While the change in EQ-5D-5 L at one-year post-operative was statistically greater in the raTKA cohort, the difference between groups of 0.112 did not exceed the MCID of 0.26. There were also no differences in the frequency of patients achieving MCID for pain, EQ-5D-5 L, OKS, or FJS at any time point. Our findings agree with a recent meta-analysis of prospective randomized controlled trials that also reported equivocal differences in PROM improvements between raTKA and cTKA [27]. Differences in pre-operative deformity exclusion criteria [41] and surgeon accuracy may explain some of the variance between studies. For example, a single-surgeon study of over 1400 knees over 10 years reported no difference between raTKA and cTKA in PROMs [42]. In that same study, there were no differences between cohorts for femorotibial angle, femoral or tibial component position, joint line, or posterior femoral condylar offset, which suggests good accuracy and reproducibility by the surgeon. In contrast, some [9, 43], but not all [15, 44], studies have reported an association between greater raTKA radiological accuracy and reproducibility concomitant with superior PROMs. Further studies are necessary to assess the relationship between accuracy and patient outcomes between raTKA and cTKA, as most studies have assessed each of these variables independently of one another.

Lastly, we found longer surgical times, more trays, and longer time under anesthesia in the raTKA cohort. The higher time from incision cut to incision close in the raTKA group may be explained by the time allocated for bone preparation and landmarking, implant trialing, and validation of cuts. This time recording is also reflected in the longer time under anesthesia. Although all investigators underwent training with the robotic knee system, some of the variability seen in raTKA cases may have been due to a learning curve. Van Lommel et al. [45] reported a learning curve of 11 cases, with reductions in robotic set up, bone registration, joint balancing, bone preparation and implant trialing occurring across the first 10–20 cases. Similarly, Bolam et al. [46] reported a 9-case average learning curve with no differences in operative time between cTKA and raTKA. Finally, given that Marchand et al. [47] reported a decrease in surgical times over the first year of use, continued use may lead to improved efficiency. It should be noted that this study occurred during the Covid-19 pandemic limiting the ability of the surgeons to operate in a consecutive manner, which may have also affected the learning curve.

### Limitations

This study is not without limitations. First, because this was a multi-center study, variance in surgical philosophy and techniques between surgeons may have added variability to results. However, all but one surgeon performed both raTKA and cTKA cases and rotated between raTKA and cTKA every 5 patients. To this end, there were no significant differences between the number of raTKA and cTKA performed by each surgeon, nor any differences in patient characteristics between patient cohorts pre-operatively. However, lack of randomization to raTKA and cTKA cohorts may have introduced selection bias. Second, the study was delayed due to the Covid-19 pandemic, and without a formal a priori power analysis, may have been underpowered as a result. Finally, the results of this study are limited to the specific robotic system studied herein as planning and execution varies between robotic platforms [48].

## Conclusions

In conclusion, robotic-assisted total knee arthroplasty was associated with a greater magnitude of satisfaction with the ability to perform activities of daily living at six weeks post-operative, fewer soft tissue releases and fewer cases of early post-operative medial/lateral instability, but led to equivalent clinical outcomes for overall satisfaction, quality of life, and knee-specific patient reported outcome measures in the early post-operative period. Future research, with randomized controlled studies, longer follow-up, and objective measures of function (i.e.: gait metrics) should be conducted.

## Data Availability

No datasets were generated or analysed during the current study.
